# Supplementary material for: Plasmon-induced strong interaction between chiral molecules and orbital angular momentum of light
Source: Sci Rep. 2015 Dec 14;5:18003. doi: 10.1038/srep18003 (PMC4677297; doi:10.1038/srep18003)
Supplement: Supplementary Information [file srep18003-s1.pdf]

Supplementary materials

Plasmon-induced strong interaction between chiral molecules and orbital angular momentum of light

Tong Wu, Rongyao Wang and Xiangdong Zhang

1 OAM BEAM IN APLANATIC SYSTEM

In this section, we give expressions of $\boldsymbol{E}_{inc\pm}^{(\pm l)}$ for the OAM beam in the aplanatic system. The OAM beam $\boldsymbol{E}_{inc0}^{(l)}(\rho,\varphi,z)=\left|E_{inc}^{(l)}(\rho,\varphi)\right|e^{ik_z z}\left(p_x\boldsymbol{e}_x+p_y\boldsymbol{e}_y\right)$ with LG mode focused by an object with a numerical aperture of NA can be expressed by using the vectorial Debye theory[S1-3],

$$\boldsymbol{E}_{inc+}^{(l)}(r,\theta,\phi)=\int_{\alpha=0}^{\alpha_{\max}}\int_{\beta=0}^{2\pi}\sum_{\sigma=\pm}\tilde{\boldsymbol{E}}_{\sigma}^{(l)}(\alpha,\beta)e^{ikr\left[\sin\alpha\sin\theta\cos(\beta-\phi)+\cos\alpha\cos\theta\right]}k^2\cos\alpha\sin\alpha\,\mathbf{d}\alpha\mathbf{d}\beta\,, \qquad \text{(S1-1)}$$

where $\tilde{\boldsymbol{E}}_{\sigma}^{(l)}(\alpha,\beta)$ represents the amplitude of the Fourier plane-wave spectrum, which can be written as

$$\tilde{\boldsymbol{E}}_{\sigma}^{(l)}(\alpha,\beta)=\frac{ikfe^{-ikf}}{2\pi k^2\cos\alpha}E_{\sigma}^{(l)}(\alpha,\beta)\left(\frac{\boldsymbol{e}_\alpha+i\sigma\boldsymbol{e}_\beta}{\sqrt{2}}\right), \qquad \text{(S1-2)}$$

and

$$E_{\sigma}^{(l)}(\alpha,\beta)=\frac{1}{\sqrt{2}}\left|E_{inc}^{(l)}(f\sin\alpha,\beta)\right|\sqrt{\frac{n_1}{n_2}}\cos^{1/2}\alpha\left[v\mp i\sigma u\right]\ , \qquad \text{(S1-3)}$$

$$\left|E_{inc}^{(l)}(\rho,\varphi)\right| = \left[\frac{\sqrt{2}\rho}{\omega_0}\right]^{|l|} \exp\left[-\frac{\rho^2}{\omega_0^2}\right] L_p^{|l|}\left(-\frac{\rho^2}{\omega_0^2}\right) e^{il\varphi}, \tag{S1-4}$$

$$v = \left[p_y \sin\beta + p_x \cos\beta\right], \tag{S1-5}$$

$$u = \left[p_y \cos\beta - p_x \sin\beta\right]. \tag{S1-6}$$

Here k_z is the z component of the wave vector, (p_x, p_y) = (1, 0) or (0, 1) describe linearly-polarized beams, and $(p_x, p_y) = (1, \pm i)$ represents, respectively, left-circularly and right-circularly polarized beam. Here f is the focal distance of lens while ω_0 is the beam waist radius before focusing, $\alpha_{\max}$ is related to the numerical aperture through $NA = n_2 \sin\alpha_{\max}$, n_1 and n_2 are the refractive indices in the object and imagine regions, respectively. $L_p^l(x)$ is the associated Laguerre polynomials. At the same time $\boldsymbol{e}_\beta = -\sin\beta \boldsymbol{e}_x + \cos\beta \boldsymbol{e}_y$, and $\boldsymbol{e}_\alpha = \cos\alpha\cos\beta\boldsymbol{e}_x + \cos\alpha\sin\beta\boldsymbol{e}_y - \sin\alpha\boldsymbol{e}_z$. The field is now expanded into series of VSFs as the form given by the following relation:

$$\boldsymbol{E}_{inc+}^{(l)}(\boldsymbol{r}) = \sum_{v=1}^{\infty} a_{v+}^{(l)} \boldsymbol{M}_v^{(1)}\left(k\left(\boldsymbol{r}-\boldsymbol{r}_d\right)\right) + b_{v+}^{(l)} \boldsymbol{N}_v^{(1)}\left(k\left(\boldsymbol{r}-\boldsymbol{r}_d\right)\right). \tag{S1-7}$$

The $\boldsymbol{M}_v^{(1)}$ and $\boldsymbol{N}_v^{(1)}$ are VSFs of the first kind whose definition can be found in ref S4. Here $\boldsymbol{r}_d = (\rho_0\cos\phi_0, \rho_0\sin\phi_0, z_0)$ is the position of the chiral molecule, and the expansion coefficients should be written as[S3,S5]

$$\begin{aligned}\begin{bmatrix} ia_{v+}^{(l)} \\ b_{v+}^{(l)} \end{bmatrix} = {} & 2\eta_f i^n e^{i(l-m)\phi_0}\left[\gamma_{mn}\frac{n_1}{n_2}\right]^{1/2} \operatorname{sgn}^m(1,m)\int_0^{\alpha_{\max}} d\alpha\sqrt{\cos\alpha}\sin\alpha e^{ikz_0\cos\alpha}\,\mathsf{P}_{pl}(\sin\alpha)e^{-f^2\sin^2\alpha/\omega_0^2} \\ & \times\left\{\left[\frac{(m-l)J_{m-l}(k\rho_0\sin\alpha)\pi_{mn}(\cos\alpha)}{k\rho_0\sin\alpha} + J'_{m-l}(k\rho_0\sin\alpha)\tau_{mn}(\cos\alpha)\right]\begin{bmatrix} -p_x\cos\phi_0 - p_y\sin\phi_0 \\ -p_y\cos\phi_0 + p_x\sin\phi_0 \end{bmatrix}\right. \\ & \left. + i\left[\frac{(m-l)J_{m-l}(k\rho_0\sin\alpha)\tau_{mn}(\cos\alpha)}{k\rho_0\sin\alpha} + J'_{m-l}(k\rho_0\sin\alpha)\pi_{mn}(\cos\alpha)\right]\begin{bmatrix} -p_x\sin\phi_0 + p_y\cos\phi_0 \\ -p_y\sin\phi_0 - p_x\cos\phi_0 \end{bmatrix}\right. \end{aligned} \tag{S1-8}$$

with

$$\mathsf{P}_{pl}(\sin\alpha) = -\left[\frac{\sqrt{2}f\sin\alpha}{\omega_0}\right]^{|l|} L_p^{|l|}\left(\frac{2f^2\sin^2\alpha}{\omega_0{}^2}\right), \tag{S1-9}$$

$$\pi_{mn}(\cos\alpha) = \frac{m}{\sin\theta}P_n^m(\cos\theta) \quad , \tag{S1-10}$$

$$\tau_{mn}(\cos\alpha) = \frac{d}{d\theta}P_n^m(\cos\theta) \quad , \tag{S1-11}$$

where η_f is expressed as $\eta_f = ikfe^{-ikf}$, and $\gamma_{mn} = \frac{(2n+1)(n-m)!}{n(n+1)(\mathrm{n}+m)!}$. At the same time, $\pi_{mn}(\cos\alpha) = \frac{m}{\sin\theta}P_n^m(\cos\alpha)$ and $\tau_{mn}(\cos\alpha) = \frac{d}{d\theta}P_n^m(\cos\alpha)$, with $P_n^m(\cos\alpha)$ being the associated Legendre functions. $\mathsf{P}_{pl}(\sin\alpha)$ is a function related to the associated Laguerre polynomials, whose expression is given by

$$\mathsf{P}_{pl}(\sin\alpha) = \left[\frac{\sqrt{2}f\sin\alpha}{\omega_0}\right]^{|l|} L_p^{|l|}\left(\frac{2f^2\sin^2\alpha}{\omega_0^2}\right). \tag{S1-12}$$

Without loss of generality, here we limit our discussion on x polarized waves with $(px, py) = (1,0)$, and fix the beam center at the origin of the coordinate. $\boldsymbol{E}_{inc-}^{(-l)}(\boldsymbol{r})$ is defined as the electric field which is related to $\boldsymbol{E}_{inc+}^{(l)}(\boldsymbol{r})$ through the space inversion at $\boldsymbol{r}_d$, which is still an OAM beam but possessing a topological index of $-l$.

$$\boldsymbol{E}_{inc-}^{(-l)}(\boldsymbol{r}) = \left[\hat{P}\boldsymbol{E}_{inc+}^{(l)}\right]. \tag{S1-13}$$

Here $\hat{P}$ is the parity operator. The minus sign in the subscript represents the direction of propagation, which is opposite with that of $\boldsymbol{E}_{inc+}^{(l)}$. If $\boldsymbol{E}_{inc-}^{(-l)}(\boldsymbol{r})$ is expressed as form of sum of VSFs functions like Eq.(S1-7), the expansion coefficients are written as

$$a_{\nu-}^{(-l)} = (-1)^{n+1} a_{\nu+}^{(+l)}; b_{\nu-}^{(-l)} = (-1)^n b_{\nu+}^{(+l)}. \tag{S1-14}$$

2 VALUES OF INTEGRATIONS [S6]

In this section, we give the expressions for $I_1^{(ml)}, I_2^{(ml)}, I_3^{(ml)} = -I_2^{(ml)}$ and $I_4^{(ml)}$.

$$\begin{aligned} I_1^{(ml)} &= (m-l)^2 \int_0^\infty \frac{J_{m-l}(k\rho_0 \sin\alpha) J_{m-l}(k\rho_0 \sin\alpha')}{k^2 \rho_0^2 \sin\alpha \sin\alpha'} \rho_0 d\rho_0 \\ &= \begin{cases} \frac{1}{2k^2}(m-l)\frac{\sin^{m-l-1}\alpha}{\sin^{m-l+1}\alpha'} & (\sin\alpha < \sin\alpha') \\ \frac{1}{2k^2}(m-l)\frac{\sin^{m-l-1}\alpha'}{\sin^{m-l+1}\alpha} & (\sin\alpha \geq \sin\alpha') \end{cases} , \end{aligned} \tag{S2-1}$$

$$I_2^{(ml)} = (m-l)\int_0^\infty \frac{J_{m-l}(k\rho_0 \sin\alpha) J'_{m-l}(k\rho_0 \sin\alpha')}{k \sin\alpha} d\rho_0 = \begin{cases} -I_1^{(ml)} & (\sin\alpha < \sin\alpha') \\ I_1^{(ml)} & (\sin\alpha \geq \sin\alpha') \end{cases}, \tag{S2-2}$$

$$\begin{aligned} I_4^{(ml)} &= \int_0^\infty J'_{m-l}(k\rho_0 \sin\alpha') J'_{m-l}(k\rho_0 \sin\alpha) \rho_0 d\rho_0 \\ &= \int_0^\infty J_{m-l+1}(k\rho_0 \sin\alpha') J_{m-l+1}(k\rho_0 \sin\alpha') d\rho_0 + I_1^{(ml)} + \hat{I}_2^{(ml)} + \hat{I}_2^{(ml)}{}', \\ &= \frac{1}{k \sin\alpha} \delta(k \sin\alpha - k \sin\alpha') + I_1^{(ml)} + \hat{I}_2^{(ml)} + \hat{I}_2^{(ml)}{}' \end{aligned} \tag{S2-3}$$

References

[S1] Novotny, L.; Hecht, B. *Principles of Nano-Optics*, (University Press:Cambridge, 2006).

[S2] Monteiro, P. B., Neto, P. A. M., Nussenzveig, H. M. Angular momentum of focused beams: Beyond the paraxial approximation. *Phys. Rev. A* **79**, 033830 **(**2009).

[S3] Ng, J., Lin, Z., Chan, C. T. Theory of optical trapping by an optical vortex beam. *Phys. Rev. Lett.* **104**, 103601 (2010).

[S4] Doicu, A., Wriedt, T., Eremin, Y. A. *Light Scattering by Systems of Particles*,

(Springer-Verlag, Berlin, 2006).

[S5] Alvaro, R. N. A. *et al*. Exact partial wave expansion of optical beams with respect to an arbitrary origin. *Opt. lett.*, **31**, 2477-2479 (2006**)**.

[S6] Watson, G. N. A Treatise on the Theory of Bessel Functions, (Cambridge University Press, Cambridge, 1980).
